# Successful Outcome With a Multimodal Approach in the Management of Pediatric Necrotizing Fasciitis: A Report of a Rare Case

**DOI:** 10.7759/cureus.50807

**Published:** 2023-12-19

**Authors:** Rajesh Domakunti, Kiran Khedkar, Varun Kulkarni

**Affiliations:** 1 Department of General Surgery, Jawaharlal Nehru Medical College, Datta Meghe Institute of Medical Sciences, Wardha, IND

**Keywords:** pediatric sepsis, pediatric fractures, skin grafting, serial dressings, emergency debridement

## Abstract

Necrotizing fasciitis is a commonly encountered modality by a practicing clinician which needs urgent and vigilant management. To decrease the potential morbidity and mortality of the pathology, an aggressive multimodal approach shows promising results. In our report, we present a case of a three-year-old male child who presented to us with the findings consistent with necrotizing fasciitis. The case report highlights the importance of multimodal approach in such a case.

## Introduction

Clinicians frequently deal with necrotizing fasciitis (NF), a potentially fatal infective condition of the soft tissue. It is characterised by rapidly progressive fascial and subcutaneous necrosis. The prognosis is greatly influenced by the attending surgeon's attentiveness and prompt diagnosis [1]. According to Puri and Innes, NF is essentially a severe inflammation of the muscle sheath that leads to necrosis of the subcutaneous tissue and adjacent fascia [1]. It is difficult to diagnose early and even more difficult to manage effectively [2]. Early clinical suspicion, appropriate antimicrobials, and surgery are key to improving survival as stated in one of the surveys of the invasive group [3]. A beta-hemolytic streptococcal (GAS) infection, including NF, the correct diagnosis was initially suspected in only 2% of admissions [4].

Thus, the clinical presentation of the pathology needs to be attended very carefully to make a proper diagnosis. Simultaneous aggressive management along with its diagnosis is also crucial in this type of cases. A multimodal approach towards disease management has a significant role in decreasing the morbidity and mortality of the condition and decreasing the disease burden on society.

## Case presentation

A three-year-old male child was presented to the emergency department with a chief complaint of severe pain and blackening of skin over the left thigh for one day, as per the history narrated by the child's father. A detailed history from the child's relatives revealed a fall from a bike 10 days ago with injuries over the forehead and left thigh, following which he could not walk. He was taken to an unprincipled quack nearby, where the left leg was immobilised by the application of unauthorised lotion or ointment made from various leaves and other non-documented ingredients over the entire left thigh with support from multiple wooden sticks covering it. It was not opened for around eight days. As the child developed severe pain over the site, it was opened, and blackening over the covered area was noted. The child was then presented to our Institute for further evaluation and management.

On a general physical examination, the patient was irritable, disoriented, and febrile. On local examination of the left thigh, a deformed leg with multiple blisters and necrosed blackish skin covering the entire circumference of the thigh was noted. It also had foul-smelling, purulent discharge from the lesion. A diagnosis of NF was made for the patient. A limited range of movement was seen at the hip joint and knee joint. An X-ray of the left thigh was done, which revealed a fractured shaft of the left femur. Immobilisation of the involved leg was done by applying traction with an appropriate weight for the child's age as per the orthopaedic surgeon's advice.

**Figure 1:**
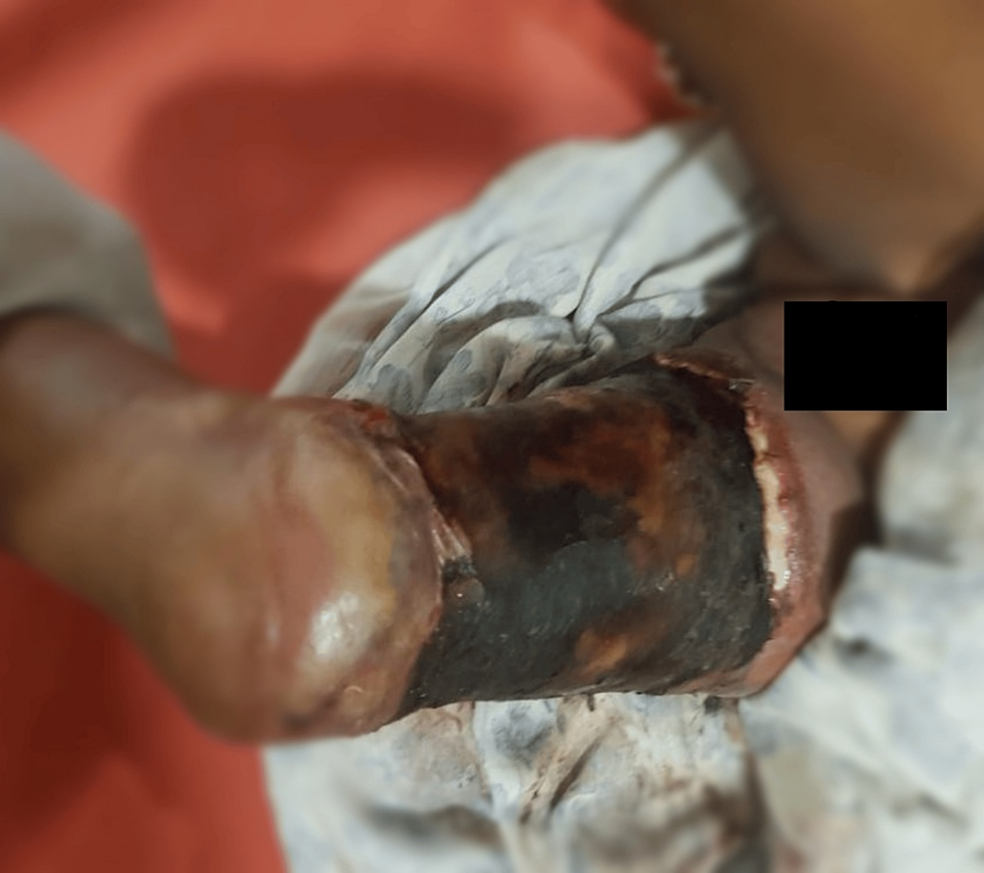
The wound over the leg of the patient

The patient was admitted under the Department of Pediatrics and kept nil per oral till further evaluation and management. All routine laboratory investigations were sent which reported values within normal limits except the white blood cell (WBC) count. The total leukocyte count was raised to 24,900 cells/mm^3^, suggesting severe sepsis (Figure 1).

**Figure 2:**
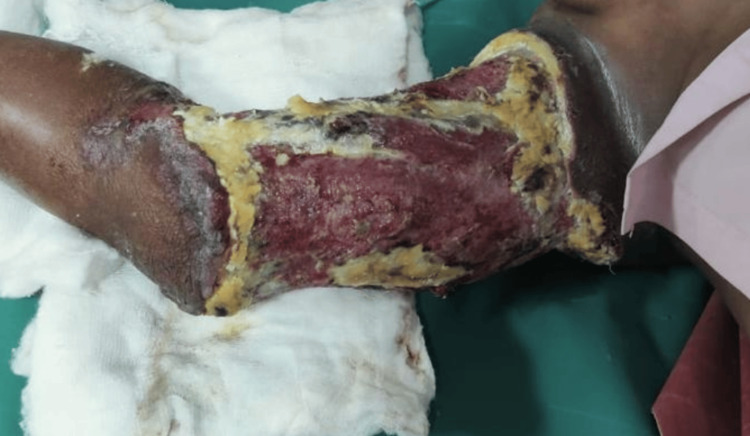
Post-debridement wound

On the second day of admission, the patient was taken for emergency surgery under general anaesthesia. Mechanical debridement of the necrosed skin over the thigh was done, and pus was sent for culture sensitivity. The pus culture report revealed the growth of multiple organisms, including *Escherichia coli*, *Pseudomonas aeruginosa*, and *Klebsiella pneumoniae*. Thus, the patient was then managed conservatively with a daily bedside sterile dressing with betadine. Serial debridement procedures under mild sedation and under cover of appropriate injectable antibiotics as per the sensitivity reports of the microorganisms obtained from the pus culture were performed. The patient was also given sufficient blood transfusions to optimise the haemoglobin levels, which were reduced due to blood loss during surgery and daily debridement. This continued until most of the debris were removed from the wound area (Figure 2).

**Figure 3:**
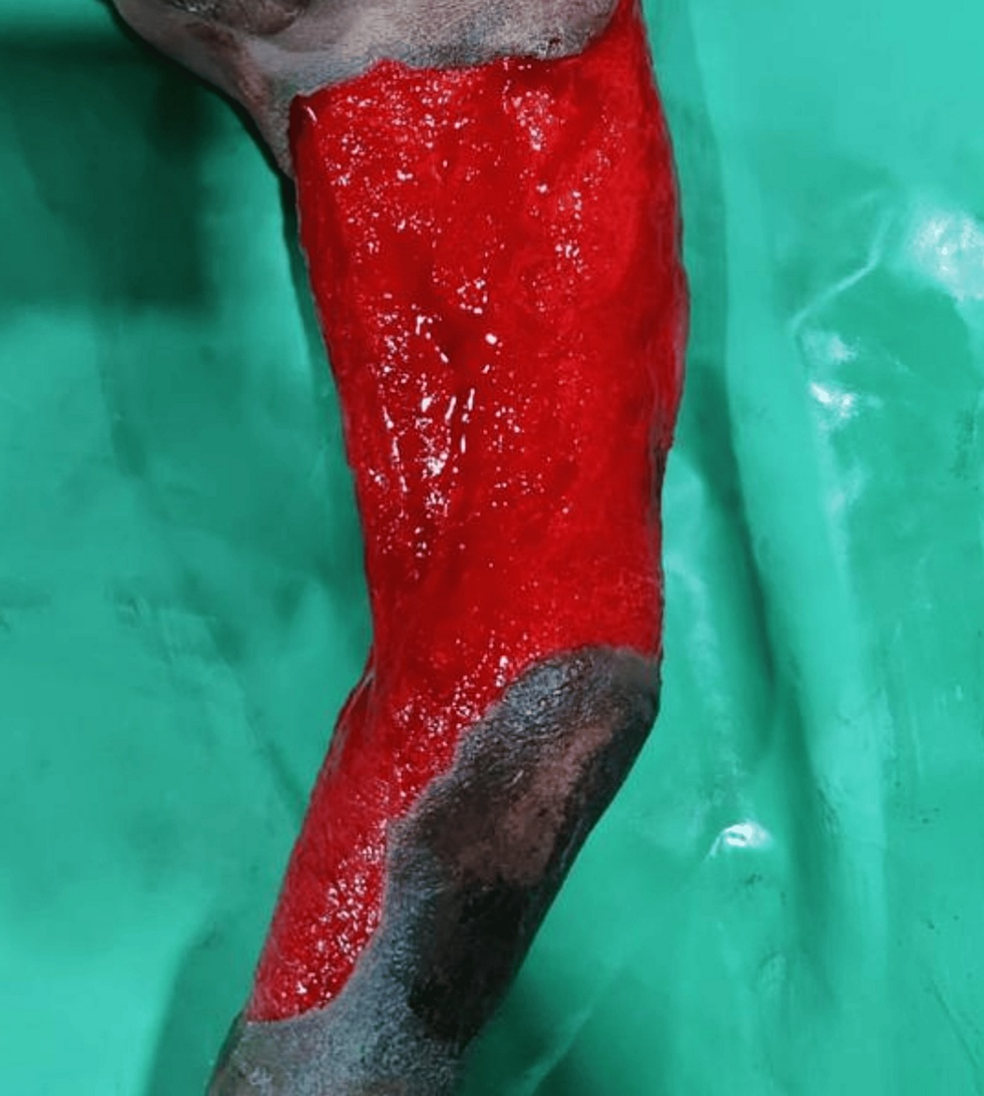
Healthy granulation tissue after vacuum-assisted dressing

On the 12th day of admission, the plastic surgeon's opinion was taken for further management of the wound, for which negative pressure wound therapy (NPWT) (vacuum-assisted closure (VAC) dressing of the wound) followed by partial-thickness skin grafting was advised. Hence, as per their advice, VAC dressing was done for two sittings, along with the application of Cadomer (JB Chemicals & Pharmaceuticals Ltd., Mumbai, India) and collagen granules, keeping each dressing for five days. This showed a rapid progression in wound healing and the generation of healthy granulation tissue over the entire debrided area (Figure 3).

**Figure 4:**
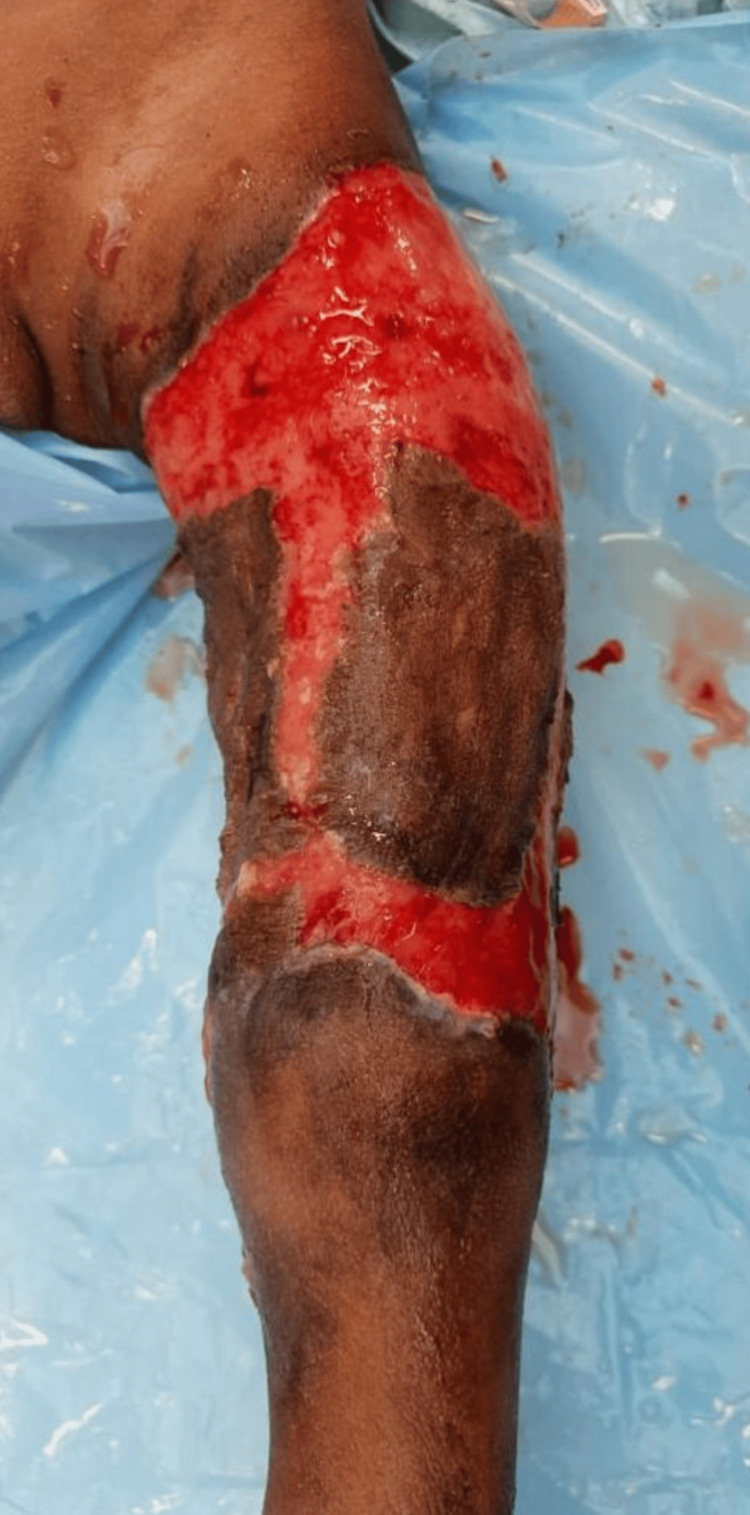
Post-skin grafting status

On the 25th day of admission, the patient underwent a partial-thickness skin grafting procedure under general anaesthesia. However, because of the smaller body surface area, the entire area was not covered with skin grafts even after meticulous meshing. The rest of the wound was left open to heal by secondary intention. The patient was kept under strict observation for five days postoperatively to watch for any signs of graft rejection. However, after opening the dressing, it was noted that the graft was taken. Throughout the course in the hospital, the thigh was immobilised with traction application to manage the fracture of the femur conservatively. Hence, the patient was discharged after the removal of traction as per the orthopaedic surgeon's advice. The patient was regularly followed up after that until the complete healing of the wound (Figure 4).

## Discussion

NF involving the limbs is a comparatively rarely encountered infective condition of the subcutaneous tissue. It rapidly spreads along fascial planes [5]. Because of the destruction of the fascial tissue, this condition can turn out to be fatal [6-8]. Timely diagnosis and urgent, aggressive management from a surgical and intensive care point of view are deciding factors in the prognosis and outcome of the patient. Even though recent times have brought up many advanced methods of surgical, medical, and critical care management, the mortality rate remains high, exceeding the mark of 75% [9,10]. Recent reports of improved results, with a mortality rate below 10%, by certain authors raise the possibility that things are getting better [11]. The mortality rate for NF remained on the higher side (30%), and the complications developed by surviving patients were also significant in a cohort of 20 patients treated in a high-tech tertiary centre [11]. To the best of our knowledge, recent developments in wound care, such as NPWT and hydro-bisturi-assisted debridement (HAD), have not shown significant benefits in cases of NF. A number of studies have reported that a patient with NF may suffer from a longer hospital stay, more debridement, limb loss, or even death if their diagnosis is delayed [12,13]. Regretfully, it might be challenging to diagnose NF. A delay in diagnosis characterises this form of infection because of the feature of developing along the fascia and the absence of evident initial pathognomonic indications [5,7,14]. In an attempt to differentiate NF from other soft tissue diseases, Wong et al. created the Laboratory Risk Indicator for Necrotizing Fasciitis (LRINEC), which uses analytical indicators like blood sodium, creatinine, haemoglobin, WBC count, and glucose levels. The authors state that the likelihood of NF is <50% for scores ≤5, 50-75% for scores of 6 or 7, and >75% for scores ≥8 (lowest score of 0 and maximum score of 13) [10]. The system hasn't been thoroughly validated, as far as we know. Early identification and management of NF are crucial because the mortality of NF increases with a delay in diagnosis and proper treatment.

Repeated clinical evaluation, a multiparametric approach combining a variety of diagnostic modalities, and multidisciplinary participation will optimise the diagnosis in situations where the diagnosis is unclear. As soon as the diagnosis is entertained, infection control measures should be considered, and antimicrobial therapy should be adjusted according to the infecting organism. Although a "second look" is advised, early surgical referral is crucial for therapeutic excision of as much diseased tissue as feasible and diagnostic confirmation.

## Conclusions

The unavailability of nearby medical health facilities and the illiteracy of the child's parents made them visit the quack for treatment of illness. This approach further worsened the child's condition and led to the development of NF. Once the patient reached the hospital, he was treated with multimodal approach. It included mechanical debridement, application of various chemical agents like cadexomer (Cadomer) collagen granules, NPWT, and partial-thickness skin grafting. It was supplemented with appropriate antibiotic coverage and maintenance of strict aseptic measures. This required a broad scientific knowledge regarding pediatric NF, newer wound healing techniques, skin grafting, immense patience, and skilled and experienced surgical hands. It is a very challenging task practically, as NF is an uncommon entity in the pediatric age group. It carries a high risk of mortality if not treated aggressively and appropriately as early as possible. However, in our Institute, the involvement of a multidisciplinary team containing a pediatric surgeon, pediatricians, general surgeons, plastic surgeons, orthopaedic surgeons, and anaesthetists made us overcome all the odds and achieve a fruitful outcome. Thus, the patient was discharged with a complete cure and the least possible disability.

There is a famous proverb, "Prevention is better than cure". If this strategy is applied to the current case, then increasing the awareness regarding modern medical science, making the healthcare facility accessible in remote areas for the public, standardising the laws against such illegal medical practitioners, and improving the current literacy of the Indian population would reduce such incidence in the upcoming future. We present a rare case of pediatric NF, which was treated successfully with a multidisciplinary approach. We conclude that this multidisciplinary approach helps in a better outcome reducing the morbidity and mortality burden on society.
